# Commentary: Identification of Mutation Regions on *NF1* Responsible for High- and Low-Risk Development of Optic Pathway Glioma in Neurofibromatosis Type I

**DOI:** 10.3389/fgene.2019.00115

**Published:** 2019-03-01

**Authors:** Corina Anastasaki, Feng Gao, David H. Gutmann

**Affiliations:** ^1^Department of Neurology, Washington University School of Medicine, St. Louis, MO, United States; ^2^Department of Surgery, Washington University School of Medicine, St. Louis, MO, United States

**Keywords:** neurofibromatosis type 1, genotype-phenotype correlations, optic pathway glioma, mutation location, clinical risk assessment

As we begin to consider risk assessment strategies critical to the implementation of precision medicine, it becomes important to identify and evaluate clinically actionable genotype-phenotype associations. Germane to the future management of children with the tumor predisposition syndrome, Neurofibromatosis type 1 (NF1; MIM #162200), the recent report by Xu et al. demonstrates a positive correlation between optic pathway glioma (OPG) and *NF1* gene mutation location (Xu et al., 2018). In clinical practice, only 15–20% of these at-risk children with NF1 develop OPGs, low-grade astrocytic tumors of the optic nerve, chiasm, tracts and radiations, which can lead to visual, endocrine, and neurological deficits (Listernick et al., 2007). Currently, the lack of biomarkers predictive of OPG development limits our ability to provide prognostic information to these children and their families.

One such clinically actionable biomarker is the germline *NF1* gene mutation. While NF1 is caused by a germline mutation in the *NF1* gene, the large number of unique *NF1* mutations identified, as well as their lack of spatial clustering, has led to the conclusion that all *NF1* mutations are functionally identical. This notion of “mutational equivalency” has been recently challenged by converging data from population-based (Upadhyaya et al., 2007; Pinna et al., 2015; Anastasaki et al., 2017; Kehrer-Sawatzki et al., 2017; Koczkowska et al., 2018a,b; Morris and Gutmann, 2018), human induced pluripotent stem cell (Anastasaki et al., 2015), and genetically engineered mouse studies (Li et al., 2016; Toonen et al., 2016), which have each demonstrated striking differences in the effects of the germline *NF1* gene mutation on clinical phenotypes. For instance, large genomic deletions and missense mutations in codons 844-848 are associated with more severe clinical phenotypes in NF1 patients (Kehrer-Sawatzki et al., 2017; Koczkowska et al., 2018b). Conversely, patients with mutations Arg1809Cys and Met992del do not develop dermal and plexiform neurofibromas (Upadhyaya et al., 2007; Pinna et al., 2015).

Adding to these findings, Xu et al. identified two regions of *NF1* mutation clustering: (1) the cysteine/serine-rich domain (CSRD, residues 543–909), which was positively associated with OPG, and (2) the HEAT-like repeat region (HLR, residues 1,825–2,428), which negatively correlated with OPG. Additionally, the authors conclude that patients with OPGs were not more likely to harbor mutations within the 5′-tertile of the *NF1* gene. Their elegant analysis prompted us to extend our prior analyses using a larger series of previously published and clinically-characterized cohorts of patients with NF1, including their subjects, now with sufficient statistical power to detect potential genotype-phenotype correlations.

To determine whether mutations within specific predicted functional regions were associated with a higher risk of OPG, we employed a combined cohort of 310 NF1 patients who underwent brain magnetic resonance imaging and harbored known *NF1* gene (Anastasaki et al., 2017). Using these radiographic criteria, 101 patients harbored an OPG (33%) and 209 did not (67%; no OPG at ≥10 years of age). We then segmented the *NF1* gene into nine regions, as described by Xu et al. (CSRD, TBD, GRD, Sec14/PH, HLR, NLS, SBR, CTD and “other” to signify the six regions in between the predicted functional domains), and scored the number of patients with a germline mutation in each region. A separate χ^2^ test (2 × 2 table) was performed at each of these segments to compare the rates of mutation between patients with and without OPG. As such, we calculated the odds ratios (OR) of presenting with an OPG when the *NF1* germline mutation falls within one of the nine described regions, and additionally analyzed the associated sensitivity and specificity for each region. We did not compare each of the regions with the rest of the eight regions. The objective of the manuscript was to confirm associations suggested by previous studies by analyzing the candidate hypotheses, rather than by performing a completely new exploratory analysis. In this regard, we were more concerned about type II errors, especially given the limited sample size caused by the relative rareness of the disease. An adjustment, such as the Bonferroni correction, would reduce type I errors at the expense of increasing type II errors (Rothman, 1990, 2014). Therefore, we did not adjust the resultant *p*-values for multiple comparisons. Using this approach, we confirmed that patients with OPG in this larger cohort were more likely to harbor mutations within the CSRD domain (OR 2.05; *p* = 0.028). Conversely, NF1 patients were less likely to develop an OPG if they harbored mutations within the HLR domain (OR = 0.36, *p* = 0.013) (Table 1A). Importantly, this finding persists (CSRD: OR = 2.21, *p* = 0.005; HLR: OR = 0.4, *p* = 0.009) when the analysis was repeated to include the additional 26 OPG (total *n* = 127) and 45 no-OPG (total *n* = 254) patients from the Xu study (Table 1B), as well as when the analyses were only performed to include truncating *NF1* mutations (data not shown).

**Table 1:** Risk of OPG varies with mutations in different regions of the *NF1* gene.

**(A)**							
**Region (aa)**	**OPG** ***n*** **(%)** ***N*** **=** **101**	**no-OPG** ***n*** **(%)** ***N*** **=** **209**	**Sensitivity**	**Specificity**	**OR**	**95% CI**	***P*****-value**
CSRD (543–909)	22 (21.78)	25 (11.96)	0.22	0.88	2.05	1.09–3.86	0.028
TBD (1,095–1,197)	4 (3.96)	14 (6.7)	0.04	0.93	0.57	0.18–1.79	0.339
GRD (1,198–1,530)	13 (12.87)	29 (13.88)	0.13	0.86	0.92	0.45–1.85	0.809
Sec/PH (1,560–1,816)	7 (6.93)	11 (5.26)	0.07	0.95	1.34	0.50–3.57	0.558
HLR (1,825–2,428)	8 (7.92)	40 (19.14)	0.08	0.81	0.36	0.16–0.81	0.013
NLS (2,534-2,550)	0 (0)	0 (0)	n/a	n/a	n/a	n/a	n/a
SBR (2,619–2,719)	1 (0.99)	7 (3.35)	0.01	0.97	0.29	0.35–2.38	0.248
CTD (2,260–2,817)	9 (8.91)	23 (11)	0.09	0.89	0.79	0.352–1.78	0.571
Other	46 (45.54)	83 (39.71)	0.46	0.6	1.27	0.79–2.05	0.39
**(B)**							
**Region (aa)**	**OPG** ***n*** **(%)** ***N*** **=** **127**	**no-OPG** ***n*** **(%)** ***N*** **=** **254**	**Sensitivity**	**Specificity**	**OR**	**95% CI**	***P*****-value**
CSRD (543–909)	29 (22.83)	30 (11.81)	0.23	0.88	2.21	1.26–3.88	0.005
TBD (1,095–1,197)	4 (3.15)	15 (5.91)	0.03	0.94	0.52	0.17–1.59	0.252
GRD (1,198–1,530)	16 (12.6)	38 (14.96)	0.13	0.85	0.82	0.44–1.53	0.534
Sec/PH (1,560–1,816)	10 (7.87)	13 (5.12)	0.08	0.95	1.58	0.67–3.72	0.291
HLR (1,825–2,428)	11 (8.66)	49 (19.29)	0.09	0.81	0.4	0.2–0.8	0.009
NLS (2,534–2,550)	0 (0)	0 (0)	0.01	1	n/a	n/a	n/a
SBR (2,619–2,719)	1 (0.79)	8 (3.15)	0.02	0.96	0.44	0.09–2.05	0.292
CTD (2,260–2,817)	12 (9.45)	27 (10.63)	0.13	0.87	1.07	0.57–2.02	0.829
Other	56 (44.09)	101 (39.76)	0.44	0.6	1.19	0.78–1.84	0.418
**(C)**							
**Region (aa)**	**OPG** ***n*** **(%)** ***N*** **=** **101**	**no-OPG** ***n*** **(%)** ***N*** **=** **209**	**Sensitivity**	**Specificity**	**OR**	**95% CI**	***P*****-value**
1 (1–939)	61 (60.4)	82 (39.23)	0.6	0.61	2.36	1.45–3.84	<0.001
2 (940–1,878)	27 (26.73)	81 (38.76)	0.27	0.61	0.58	0.34–0.97	0.038
3 (1,879–2,817)	13 (12.87)	46 (22.01)	0.13	0.78	0.52	0.27–1.02	0.058
**(D)**							
**Region (aa)**	**OPG** ***n*** **(%)** ***N*** **=** **127**	**no-OPG** ***n*** **(%)** ***N*** **=** **254**	**Sensitivity**	**Specificity**	**OR**	**95% CI**	***P*****-value**
5′ (1–939)	74 (8.27)	100 (39.37)	0.58	0.61	2.15	1.39–3.32	<0.001
Middle (940–1,878)	35 (27.56)	124 (38.19)	0.28	0.51	0.4	0.25–0.63	<0.001
3′ (1,879–2,817)	18 (14.17)	57 (22.44)	0.14	0.78	0.57	0.32–1.02	0.058

***(A–D)** Tables summarize the specificity, sensitivity and odds ratios of OPG when the NF1 mutation occurs **(A,B)** within specific predicted neurofibromin functional domains or **(C,D)** within each tertile of the NF1 gene. Analysis of the Anastasaki cohort **(A,C)** and the combined Anastasaki and Xu cohorts **(B,D)**. Of note, the CTD encompasses the HLR, NLS and SBR domains. Statistically significant values appear in bold. OPG, optic pathway glioma; OR, odds ratios*.

We next segmented the *NF1* gene into three tertiles (5′-tertile, residues 1–939; middle tertile, residues 940–1,878; 3′-tertile, residues 1,879–2,817), and performed similar χ^2^ OR analyses to establish whether there was mutational clustering in the 5′-tertile of the *NF1* gene in patients with OPG. We have previously reported that cohorts smaller than 307 patients are not sufficiently powered to detect differences in mutational localization across the *NF1* locus (Anastasaki et al., 2017). As such, children with OPGs were more likely to harbor mutations in the 5′-tertile in the Anastasaki dataset only (OR = 2.36, *p* < 0.001, *n* = 310; Table 1C) or in the combined Anastasaki and Xu dataset (OR = 2.15, *p* < 0.001, *n* = 381; Table 1D), but not in the Xu dataset alone (*n* = 215). Moreover, mutations in the middle tertile of the *NF1* gene were associated with a lower risk of OPG both in the Anastasaki dataset (OR = 0.58, *p* = 0.038) and in the combined dataset (OR = 0.4; *p* < 0.001), albeit with low sensitivity (0.27 and 0.28, respectively) (Tables 1C,D).

Together with the new data presented herein, the findings reported by Xu et al. have several important implications. First, they underscore the importance of studying a large clinical population in order to generate sufficient statistical power to discover accurate genotype-phenotype associations. Second, they validate the 5′-tertile mutation clustering in NF1 patients with OPG, with mutations preferentially occurring specifically within the CSRD domain. Third, individuals with NF1-OPG are less likely to harbor mutations within the HLR domain, a region associated with increased autism symptomatology (Morris and Gutmann, 2018). Fourth, with domains both upstream and downstream of the only known functional domain (RAS-GAP domain) of the *NF1* protein (neurofibromin) exhibiting differential associations with OPG development, it becomes increasingly important to explore additional biological functions for neurofibromin. This is particularly important for a disease in which RAS/RAS effector drugs have been the primary focus of targeted treatment approaches, with variable clinical efficacy. Finally, as these associations lack sufficient sensitivity and the mutation locations are only correlative, future studies should focus on multi-risk factor evaluation relevant to clinical OPG prognostic assessments for children with NF1.

## Author Contributions

CA performed the analyses; FG performed secondary statistical analyses; CA and DG wrote the manuscript.

### Conflict of Interest Statement

The authors declare that the research was conducted in the absence of any commercial or financial relationships that could be construed as a potential conflict of interest.
